# Syringic Acid Extracted from *Herba dendrobii* Prevents Diabetic Cataract Pathogenesis by Inhibiting Aldose Reductase Activity

**DOI:** 10.1155/2012/426537

**Published:** 2012-12-29

**Authors:** Xiaoyong Wei, Dan Chen, Yanchun Yi, Hui Qi, Xinxin Gao, Hua Fang, Qiong Gu, Ling Wang, Lianquan Gu

**Affiliations:** ^1^School of Basic Medical Sciences, Guangzhou University of Chinese Medicine, Guangzhou 510006, China; ^2^School of Pharmaceutical Sciences, Sun Yat-sen University, Guangzhou 510275, China

## Abstract

*Objective*. Effects of Syringic acid (SA) extracted from dendrobii on diabetic cataract (DC) pathogenesis were explored. *Methods*. Both *in vitro* and *in vivo* DC lens models were established using D-gal, and proliferation of HLEC exposed to SA was determined by MMT assay. After 60-day treatment with SA, rat lens transparency was observed by anatomical microscopy using a slit lamp. SA protein targets were extracted and isolated using 2-DE and MALDI TOF/TOF. AR gene expression was investigated using qRT-PCR. Interaction sites and binding characteristics were determined by molecule-docking techniques and dynamic models. *Results*. Targeting AR, SA provided protection from D-gal-induced damage by consistently maintaining lens transparency and delaying lens turbidity development. Inhibition of AR gene expression by SA was confirmed by qRT-PCR. IC_50_ of SA for inhibition of AR activity was 213.17 **μ**g/mL. AR-SA binding sites were Trp111, His110, Tyr48, Trp20, Trp79, Leu300, and Phe122. The main binding modes involved hydrophobic interactions and hydrogen bonding. The stoichiometric ratio of non-covalent bonding between SA and AR was 1.0 to 13.3. *Conclusion*. SA acts to prevent DC in rat lenses by inhibiting AR activity and gene expression, which has potential to be developed into a novel drug for therapeutic management of DC.

## 1. Introduction

Increasing population senescence due to improved living standards and diet has increased the incidence rate of diabetic cataract, a frequent cause of vision impairment and blindness [1]. A complex pathogenic mechanism underlies diabetic cataract. Though the exact mechanism remains uncertain, a large body of research indicates that aldose reductase (AR) is a key enzyme involved in DC development [2]. In order to reduce diabetic cataract incidence and slow the progression of diabetic cataract in current patients, further understanding of the mechanistic involvement of AR in diabetic cataract development and progression is required.

In diabetic patients, AR activation increases polyalcohol metabolism rates. As a result, glucitol accumulation in the eye caused increased osmotic pressure, alterations to cell membrane permeability, edema, and damage to cells of the optical lens. These changes block the passage of nutrients into the lens, further resulting in reduced amino acid levels accompanied by protein denaturation and polymerization. The end result of this process is cataract formation and progression [3]. Previous studies of the experimental aldose reductase inhibitors GP-1447 and KIOM-79 have demonstrated a relationship between AR reductase inhibition and prevention of diabetic cataract progression in animal models [4, 5]. Furthermore, AR deficiency has been shown to protect against sugar-induced lens opacification in rats [2], though the clinical usefulness of this observation has been limited by early clinical indicators that these drugs may also be associated with hepatic function abnormalities, gastrointestinal symptoms such as nausea and diarrhea, and skin rash or eczema [6]. Therefore, AR is contemporarily regarded as an important target for aldose reductase inhibitors (ARI) designed to prevent and treat diabetic cataract and neuropathy, though significant challenges face researchers before clinical implementation is possible [7].

Numerous contemporary reports have addressed the minimization of ARI side effects for potential treatment of diabetic cataract. Promising results have been reported using traditional Indian medicinal plants that demonstrate positive *in vitro* inhibition of aldose reductase in rat lens [8, 9]. Similar results have been observed using extracts from the Japanese Dogwood tree, *Cornus officinalis*, to inhibit AR and reduce cataract genesis *ex vivo *[10]; however, only the enzyme activity of these natural ARIs has been studied *in vitro*. Some of the most promising candidates, however, are the orchids of the *Dendrobium* genus (family Orchidaceae), used commonly in traditional Chinese medicine as dried teas and other preparations [11].

*Herba dendrobii*, known as Shi Hu in traditional Chinese medicine, is found in the stem of the orchid *Dendrobium nobile* Lindl and in many other orchid species of the *Dendrobium* genus. In ancient Chinese medical literature dating back over a thousand years, *Dendrobium* extracts were reported to improve vision [11]; however, there is still no firm understanding of the chemical components and mechanism of these extracts on vision. Recent research indicates that *Dendrobium* extracts may also have other bioactive properties, including the induction of apoptosis in human gastric cancer cells [12], the facilitation of neuroprotection against stress-induced apoptosis in PC12 cells [13], the inhibition of lipopolysaccharide- (LPS-) induced memory impairment in rats [14], and the LPS-induced nitric oxide production in macrophages [15]. It has also been suggested that *Dendrobium* extracts have strong antioxidant properties* in vitro *[16].

Syringic acid, a naturally occurring O-methylated trihydroxybenzoic acid monomer extracted from *Herba dendrobii*, has chemical properties that have been shown to facilitate oxidation, polymerization, and condensation reactions. Like most naturally occurring ARIs, syringic acid is a phenolic compound that acts pharmacologically as an antioxidant to clear free radicals [17]. These findings suggest that syringic acid may act to inhibit the development of diabetic cataract through AR.

In the present study rat cataract models established using D-galactose (D-gal) to establish were used to investigate the effects of syringic acid in treatment and prevention of diabetic cataracts both *in vitro* and *in vivo*. The mechanism of action for AR inhibition was also examined, demonstrating the novel effect of syringic acid on the pathogenesis of diabetic cataracts through experimental study.

## 2. Methods

Live specimens of* Dendrobium nobile* Lindl (Chishui Xintian *Herba Dendrobii* Co., Ltd, Guizhou, China) were acquired. The identification of species was performed by Professor Li Wei of the Guangzhou University of Chinese Medicine. Syringic acid was extracted at a purity greater than 98% using the method previously described by Zhang Xue [17].

### 2.1. Effect of Syringic Acid on Histology and Activity of Injured HLEC Induced by High-Concentration D-Gal

Human lens epithelial cell (HLEC) strain SRA01/04 (Ophthalmology Center of Sun Yat-Sen University, China) were cultured using previously published techniques [18]. During the logarithmic growth phase, cells at 10^4^ cells per well were divided into 5 groups: the normal control, the model group, the high-dose syringic acid group, the medium-dose syringic acid group, and the low-dose syringic acid group. The normal control group was cultured in Dulbecco's Modified Eagle Medium (DMEM) with 10% FBS (Gibco, USA). The model group was exposed to the same culture medium as the normal control group together with 250 mM D-gal of a purity >99% (Amresco, USA). Similarly, the high-dose, medium-dose, and low-dose syringic acid groups were exposed to D-gal and 0.4 g/L syringic acid, 0.2 g/L syringic acid, and 0.1 g/L syringic acid, respectively. HLECs were cultured for 120 h in a Galaxy-s CO_2_ incubator (RS Biotech, USA) with an atmosphere of 5% CO_2_ and temperature of 37°C. HLEC histological features were observed and photographed using a PM-inverted microscope (Olympus Company, Japan).

HLEC activity was detected using a methyl thiazolyl tetrazolium (MMT) assay (Sigma, USA). After exposure to syringic acid, cells were washed with PBS buffer solution, and 200 *μ*L of MTT (final concentration 0.5 g/L) was added to the culture wells. The cocultures were incubated in 5% CO_2_ at 37°C for 4 h. The MTT solution was discarded, and 150 *μ*L DMSO (Amresco, USA) was added. The mixed solution was mildly agitated for 10 min at room temperature, and cell growth was assayed at a wavelength of 490 nm on a fully automated ELx800 enzyme-labeled meter (BioTek, USA).

All experiments were repeated in triplicate, and results were expressed as averaged outcomes. The cell survival rate was calculated as the average absorption value of the exposed groups divided by the average absorption value of the normal control group × 100%. The cell inhibition ratio was calculated as 100% minus the cell survival rate.

### 2.2. Measuring Effects of Syringic Acid on Transparency of *In Vitro* Rat Lens

Rat lens cultures were performed according to previously published methods [19]. Briefly, rats were killed by rapid dislocation of cervical vertebrae, and the entire *Bulbus oculi* (eyeball) was extracted from each subject immediately using eye scissors. Whole specimens were washed with cold PBS (Guangzhou Genebase Biotechnology, China) containing 800 U/mL of penicillin (Guangzhou Baiyunshan Tianxin Pharmaceutical Co., Ltd., China) and 800 *μ*g/mL of streptomycin (Guangzhou Baiyunshan Tianxin Pharmaceutical Co., Ltd., China). Specimens were subsequently soaked in the same concentration of PBS solution containing a double antibody solution. Ten minutes later, specimens were transferred to a DMEM culture solution containing 500 U/mL of penicillin and 500 *μ*g/mL of streptomycin. 

The sclera was cut open from the posterior pole, and the lens was carefully removed. Following the removal of the iris and vitreous body from the surface, the lens was washed twice in PBS solution and once with DMEM solution prior to culture in preheated 24-well culture plates. Each culture well contained 1 mL of DMEM culture medium containing 20% fetal bovine serum, 100 U/mL penicillin, and 100 *μ*g/mL of streptomycin. 

After preculturing for 5 h at 37°C in a 5% CO_2_ atmosphere, visibly injured or cloudy lenses were discarded, resulting in 30 completely transparent lens for experimental use. These lenses were randomized into a normal control group (DMEM medium + 100 *μ*L FBS), a model control group (normal group medium + 250 mM D-gal), and high-, medium-, and low-dose syringic acid groups (4, 2, and 1 mg/mL syringic acid, resp.). A positive control group was soaked in D-Gal + 100 *μ*L Catalin (pirenoxine sodium) eye drops: (Batch 070102; Wuhan Tianming Pharmacy Co. Ltd., China) for 24 h.

During experiments, the culture solution was refreshed every 6 h using solutions with an unaltered D-gal concentration. Lens turbidity was evaluated by previously published methods [20]. Briefly, the lens was placed on the crosspoint of a 1 × 1 mm slide with a black checked background and observed by anatomical microscopy (Olympus, Japan). Grade 0 turbidity was indicated by clear lines, Grade I (+) turbidity was indicated by vague lines with a distinguished outline, Grade II (++) turbidity was indicated by a vague outline with indistinct centers, and Grade III (+++) turbidity was indicated by a cloudy cortex with all lines invisible. After 24 h culture, digital photos were taken of each lens (Olympus, Japan). The turbidity grade for each specimen was measured by 2 independent observers using a blind observer methodology. 

### 2.3. Measuring Effects of Syringic Acid on Transparency of *In Vivo* Rat Lens

Male and female Wistar rats aged 5-6 weeks weighing 80–120 g were provided by the Experimental Animal Center of the Guangzhou University of Chinese Medicine (China). The eyes of all subjects were pretreated with Tropicamide eyedrops (Beijing Shuanghe Pharmaceutical Co., Ltd., China). Slit lamp techniques were used to confirm lens transparency in the eighty rats selected for the experiment. Rat subjects were allowed to adaptably feed for 1 week, and they were then randomized into 4 groups stratified by body weight and gender: the normal control group and three cataract model groups (syringic acid, Calatin, and normal saline groups). Normal control group subjects received intraperitoneal injections of 10 mL/kg normal saline and were fed with drinking water and food. The cataract model was established by intraperitoneal injection of 10 mL/kg of 50% D-galactose solution twice daily, and subjects were provided with free access to 10% D-galactose solution in drinking water and food. When rat subjects demonstrated Grade I-II turbidity, they were additionally treated with either 2% syringic acid (3 drops TID, syringic acid group), Catalin eyedrops (0.8 mg/15 mL, 3 drops TID, Calatin group), or normal saline (3 drops TID, model group). Ten days later, lenses were observed by slit lamp, and turbidity was recorded as previously described. All rat subjects were observed for a full 60-day period.

### 2.4. Measurement of Syringic Acid Effects on Lens Protein Expression by 2-DE and MS

#### 2.4.1. Extraction and Detection of Total Lens Protein

Lenses were extracted from rats in the normal control, model, and syringic acid groups and placed in a mortar with lysate (1 : 10 w/v) for 1 h at room temperature. These specimens were centrifuged for 30 min at 2000 rpm at 4°C in a 5810-R low-temperature high-speed centrifuge (Eppendorf, Germany). The resulting supernatant containing the lens protein was then quantitated using the Bradford method [21], and the supernatant was preserved at −70°C.

Isoelectrofocusing (IEF) was used to isolate lens proteins. Samples (350 *μ*g) were soaked in 300*μ*L of solution with IPG Bio-Rad linear gel strips (pH 5–8) for 12–15 h at room temperature. Bio-Rad isoelectric focusing slots were used for two-dimensional electrophoresis. The gel was fixed in a stationary liquid for at least 1 h and washed three times for 10 min. The gel was then stained with Coomassie brilliant blue G-250 for 24 h. Wet stained gels were scanned with a Bio-Rad scanner, and the gel image was analyzed with the image analysis software PDQuest7.1.1 (Bio-Rad, USA).

#### 2.4.2. MALDI-TOF-TOF Mass Chromatographic Analysis and Database Search

Completely freeze-dried lens samples dissolved in 0.1% trifluoroacetic acid (TFA; Sigma, USA) were analyzed using an MS-T100LP/LC mass spectrometer (Jasco, Japan). Mass spectra were identified using a mixed spotting method. Samples (0.4 *μ*L) were spotted onto 384-well stainless steel plates, and 0.4 *μ*L of 10 mg/mL *α*-cyanocinnamic acid (CHCA; Sigma, USA) substrate solution was added. 

Matrix-assisted laser desorption/ionization (MALDI) with time of flight (TOF) analysis (MALDI TOF/TOF) was used to reveal the amino acid sequence of peptides by using postsource decay or high-energy collision-induced dissociation. Samples underwent mass-spectrometry (MS) after being allowed to naturally air dry. The MS system was comprised of a nitrogen gas laser (337 nm) using reflection technology with positive ion detection. Scans were taken at 800–4000Da using a standard peptide mixture as an external standard for calibration. The MS signal for each sample was the result of 600–800 individual laser shots. The 5 peaks with the strongest signal to noise ratio (>100) were selected from each sample as precursor ions for second-order MS analysis. The MS signal for each selected precursor ion was the result of 900–1200 individual laser shots. The resulting first-order and second-order MS were analyzed using GPS Explore V3.6 software. The first- and second-order MSs data for each sample were integrated into a single file, and proteins were identified using the MASCOT (v2.1) database.

### 2.5. Measurement of Syringic Acid Effects on AR Gene Expression by RT-PCR

#### 2.5.1. Extraction, Isolation, and Detection of Total Lens RNA

Rat lens specimens were placed on a mortar with ice and ground to form a homogenate. Homogenates were lysed in an ice bath containing Trizol (Invitrogen, USA), and total RNA was extracted. RNA purity was assayed using ultraviolet absorption assay, and RNA integrity was assayed using 1.2% formaldehyde degeneration gel electrophoresis.

#### 2.5.2. Primer and cDNA Design and Synthesis

Primer sequences of AR and *β*-actin were designed and synthesized by Shanghai Boya Biotechnological (China). The PCR upstream primer (P1) for AR was 5′-AGC GGT TTA GGT ACC ATG GGT TTT-3′, and the downstream primer (P2) was 5′-AGG GTA AGC TTC GAA TTC TCA GGC GCG GAT TTG TTG TGA-3′. The fragment length was 262 bp. P1 for the *β*-actin primer was 5′-GAGACCTTCAACACCCAGCC-3′, and P2 was 5′-GCGGGGCATCGGAACCGTCA-3′. The fragment length was 420 bp.

#### 2.5.3. PCR Analyses

Fluorescent quantitation PCR was conducted using the TaK aRa ExTaq HS enzyme (TaK, USA) and SYBR Green I fluorochrome on an ABI PRISM 7000 quantitative real-time PCR (qRT-PCR) amplifier. The PCR solution (25 *μ*L) contained Taq enzyme (12.5 *μ*L), P1 (0.5 *μ*L), P2 (0.5 *μ*L), fluorescent probe (0.5 *μ*L), the cDNA template (2.0 *μ*L), and Diethyl pyrocarbonate (DEPC) water solution (9.0 *μ*L). The amplification procedure included predegeneration at 95°C for 10 s, followed by cycle degeneration at 90°C for 5 s and at 60°C for 31 s, repeated for 40 cycles. The cycle threshold C(t) for the 40 cycles was recorded. The relative content of object mRNA copies was calculated using *α*-actin as an internal standard. Changes in gene expression level were evaluated by comparing the results from each group. The expression of the AR gene was normalized using the housekeeper gene (*β*-actin) that exhibits even expression in rat lens.

### 2.6. Measurement of AR Activity Inhibition by Syringic Acid

Aldose reductase (AR) was purchased from Prospec-Tany Technogene Ltd. (Israel). AR activity was evaluated using previously published methods [22] at a detection temperature of 25°C. The enzyme reaction solution (200 *μ*L) contained enzyme solution (20 *μ*L), 0.104 mmol/L NADPH (50 *μ*L; Italian Roth, Italy), 10 mmol/L DL-glyceraldehyde (50 *μ*L; Sigma, USA), 0.1 mol/L buffer phosphate (pH 6.2), and 4 tested concentrations (5 *μ*g/*μ*L, 10 *μ*g/*μ*L, 100 *μ*g/*μ*L, and 200 *μ*g/*μ*L) of syringic acid. The reaction was initiated by the addition of DL-glyceraldehyde substrate (Sigma, USA).

AR activity was estimated by observing the decrease of NADPH absorbance at 340 nm once every 10 s during the total 10 min reaction time. Epalrestat (Nanjing Hailing Pharmaceutical Co., Ltd., China) was used as a positive control, and PBS was used as a blank control. The AR inhibition ratio [23] was estimated as

(1) $$\begin{array}{c} \left[1-\frac{\left(A_{2}-A_{0}\right)}{\left(A_{1}-A_{0}\right)}\right]\;\times 100\%, \end{array}$$

where *A*_0_ represents the decrease in NADPH absorbance every minute without adding enzymes, substrates, or samples; *A*_1_ represents the decrease in NADPH absorbance every minute prior to sample addition; *A*_2_ represents the decrease in NADPH absorbance every minute following sample addition. The concentration of syringic acid resulting in 50% inhibition of AR (IC_50_) was determined from the resultant inhibition curves. The double-reciprocal plot method was applied for the definition of inhibitory types. The kinetic constant was calculated using the Michaelis-Menten equation.

### 2.7. Analysis of Interaction Sites and Molecular Binding Dynamics of Syringic Acid to AR

A flexible docking module (Flex X, Sybyl v.17.3) was used to identify the central region of the ligand molecule bound to the active region of the enzyme. Molecular Operating Environment Wash and Energy Minimize modules were used to study syringic acid. Gaussian03W software was used for energy reduction optimization using the basic installation (B3LYP/6-31+G(d)) [24]. An optimized minimum conformation chart was obtained, and the AR crystal structure and high-resolution structure (1USO) were obtained from the Protein Data Bank (PDB) database [25]. Using these methods, a 6.5 Å area around the ligand crystal structure was identified as the active site involved in syringic acid binding to AR.

Amber 10 Software [26] was used to create a dynamic simulation model of molecular binding of syringic acid to AR. To prepare the molecular system, topology and coordinate files were established, and the force field was set to Parm99. Three scenarios were modeled: addition of water to the syringic acid system, application of the TIP3PBOX water model, and a 9 ns gap between water and the molecule. The molecule was designed as an octahedron, thus avoiding washing the protein from the water environment. This ensured the electric neutrality of the whole system. Gaussian 03W was used for energy reduction optimization, with a basic installation set as HF/6-31G [25]. Energy optimization was completed by hydrogenation of syringic acid, coenzyme NADPH, and AR.

Molecular dynamics simulations were performed in a solvent environment at 1 atm, 300 K, 100 ps, and Tb = 2 using molecular dynamics remaining volume immobilization parameters of ntb = 1, ntp = 1, pes = 1, taup = 2, and arithmetic step width nstlim = 2500000. Molecular movement was tracked by the imaging system after dynamic simulation for 6 ns.

### 2.8. Characterization of Syringic Acid and AR Binding by Cold Spray Ionization (CSI) MS

The reserve solution contained 0.015 mmol/L dehydrated alcohol with syringic acid, and the AR reserve solution contained 2.8 *μ*mol/L of solution containing 50 *μ*g AR diluted in 500 *μ*L of water. Different volumes of syringic acid reserve solutions were placed into Ep tubes and concentrated using a pressurized gas blowing concentrator. AR solution (25 *μ*L of 2.8 *μ*mol/L) was added to each tube. Syringic acid and AR solutions at molar ratios of 3 : 1, 9 : 1, 27 : 1, 81 : 1, 243 : 1, and 729 : 1 were mixed using a vortex meter. The solutions were diluted 50-fold with 10 mmol/L ammonium acetate buffer (pH 6.7; Genebase, GeneTech Co., Ltd., China) and analyzed by MS.

The MS used a CSI source with a scan scope at m/z 100–1000. The detector voltage was 2500 V, needle voltage was −2200 V, camera lens voltage was −10 V, hole 1 voltage was −75 V, and hole 2 voltage was −8 V. The capillary tube temperature was 10°C. A volume of 20 *μ*L ammonium acetate (10 mmol/L) was automatically sampled using negative ion full scan for 1 min. 

### 2.9. Statistical Methods

Statistical analysis and data processing were completed using SPSS v.16.0 statistical software (IBM, USA). Data were expressed as means and standard deviations (mean ± SD). Intergroup differences in lens turbidity were analyzed using the Nemenyi multiple comparison *H* test. *P* values of less than were considered statistically significant (*P* < 0.05).

## 3. Results

### 3.1. Syringic Acid Affects Morphological Features and Activities of HLECs

Compared with the control group, HLECs treated with high-concentration D-gal (250 mM) became swollen, evidenced increased numbers of vacuoles and granules, and demonstrated increased numbers of irregular cells. The decrease in cell density and increased numbers of floating cells suggested the occurrence of cell death. Following exposure to different syringic acid concentrations, HLECs reverted to normal histology in different degrees. Cells exposed to the medium dosage of syringic acid recovered more completely than the both low- and high-dosage syringic acid groups (data not shown). Therefore, syringic acid appeared to reduce D-gal-induced oxidative injury in rat lens epithelial cells.

MTT assay results (Table 1) showed significantly higher HLEC inhibition (ratio 52.99%) in the model group than in other groups (*P* < 0.05). HLEC survival in the medium-dose syringic acid group (79.97%) was significantly higher than in the high-dose (71.67%) or low-dose syringic acid groups (74.38%; *P* < 0.05).

### 3.2. Effect of Syringic Acid on *In Vitro* and *In Vivo* Turbidity in Rat Lenses

Lens turbidity revealed by light microscopy after culturing for 6 h was Grade I in the model group and Grade 0 (transparent) in the other groups. After culture for 12 h, lenses were still Grade 0 in the normal control group. Simultaneously, from Grade II to III turbidity was observed in the model group, and from Grade I to II turbidity was observed in other groups (including syringic acid groups and the Catalin control).

**Figure 1:**
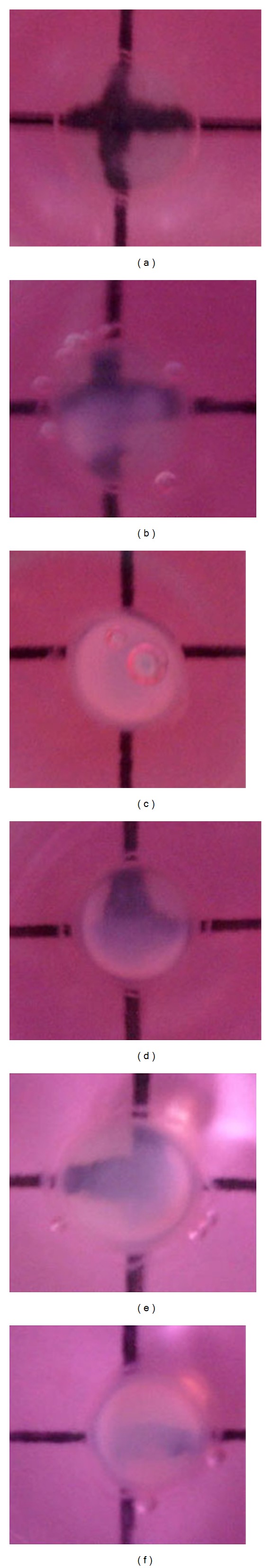
Representative photographs of lenses of rats in different groups showing effect of syringic acid on lens opacification *in vitro* (×25). (a) Control group; (b) Med-dose syringic acid group (2 mg/mL); (c) Model group; (d) Catalin group; (e) high-dose syringic acid group (4 mg/mL); (f) low-dose syringic acid group (1 mg/mL). Compared with the model group, turbidity in Catalin group and all 3 syringic acid groups was clearly reduced. Optimal effects were observed in the medium-dose syringic acid group.

 By 24 h later, all of lenses in the control group maintained Grade 0 transparency. Lenses in the model group, however, were noticeably smaller than those in the syringic acid groups. Lenses in the model group were visibly ivory-white in color, and Grade III turbidity was apparent. Lens turbidity was significantly less severe in the Catalin control group and syringic acid groups than in the model group (*P* < 0.05). Moreover, no significant differences in lens turbidity were observed between high-, medium-, and low-dose syringic acid groups and the Catalin control group (*P* > 0.05). Interestingly, medium-dose syringic acid showed the highest degree of recovery among the three syringic acid-treated groups. Cumulatively, these results suggest that syringic acid can prevent the progress of turbidity in cultured rat lenses caused by high concentrations of D-gal (*P* < 0.01; Table 2, Figure 1).

**Figure 2:**
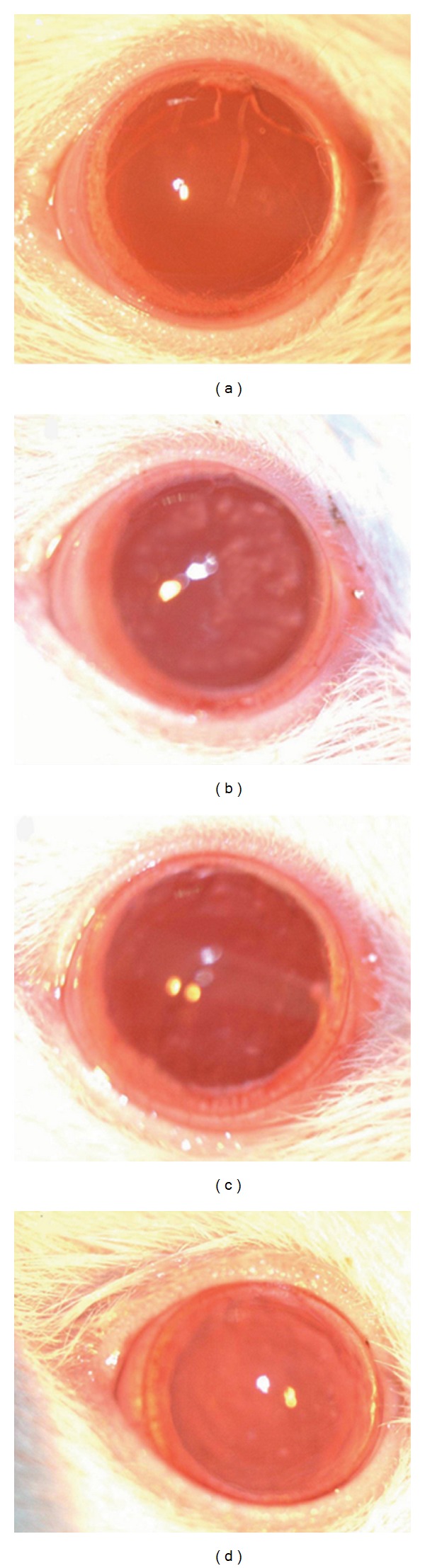
Representative photographs of the lenses of rats in different groups showing the effect of syringic acid on cataract *in vivo* (×4). (a) Control group; (b) Model group; (c) Catalin group; (d) syringic acid group. In the normal control group, lenses remained transparent. In the model group, cataracts persisted even after intraperitoneal injection of syringic acid and oral administration of D-gal had stopped (Day 90). The syringic acid groups performed better than the Catalin group, with lens turbidity reduced to Grade I or Grade 0.

Rat lenses developed cataracts 23 days after the injection of D-gal, at which point the scattered dotted turbidity around the lens became aggravated and developed from Grade II to IV turbidity (Figure 1). In the normal control group, lenses remained transparent throughout the experiment (Table 3). In the model group, cataracts persisted to day 90, even after stoppage of intraperitoneal injection of syringic acid and oral administration of D-gal. Lens turbidity began to improve after the administration of syringic acid for 10 days, further improving to Grade I or Grade 0 after 46 days of treatment. This degree of reversibility was even faster and more complete than that observed in the Catalin control group, which only achieved Grade II or Grade 0 (Figure 2).

### 3.3. Effect of Syringic Acid on Lens Differential Proteomics and Target Protein Analysis

**Figure 3:**
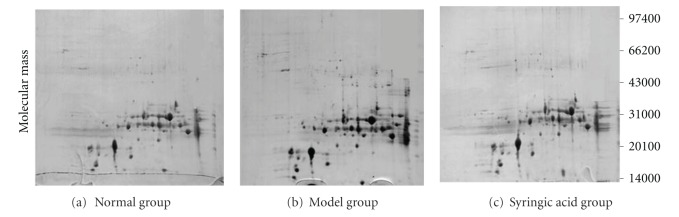
2-DE graphic of rat lens proteins. In each gel strip, spots of high-kurtosis lens protein were primarily distributed in the area of polypeptide subunits Mr 14400 to 45000 and pI 6 to 8.27. Differential protein spots were identified in the model group, showing increases compared to the normal control group (*P* < 0.01). In the syringic acid group, five spots were observed with values increased more than 2-fold compared to the model group (*P* < 0.01), and 7 differential downregulated protein spots were observed.

Two-way gel electrophoresis (2-DE) graphics of *in vivo* lens tissues are shown in Figure 3. Electrophoresis of the total lens protein from each group was repeated three times. In each gel strip, spots of high-kurtosis lens protein were primarily distributed in the area of polypeptide subunits Mr 14400 to 45000 and pI 6 to 8. One gel electropherogram was randomly selected from the 3 experimental groups, and spots of differential proteins were examined. Using automatic detection, 340 ± 6 protein spots were observed in the normal group, 427 ± 5 protein spots were observed in the model group, and 345 ± 8 protein spots were observed in the syringic acid groups. Repeatability analysis suggested that the matching rates for the 3 gel graphs were 91 ± 5%, 87 ± 6%, and 93 ± 3% in the normal, model, and syringic acid groups, respectively, indicating good intergroup repeatability.

A total of 27 differential protein spots were identified in the model group, where the relative gray value increased more than 2-fold compared to the normal control group (*P* < 0.01). In the syringic acid group, 5 spots were identified with values that increased more than 2-fold compared to the model group (*P* < 0.01). Additionally, 7 differential downregulated protein spots were identified. These 12 differential protein spots were subjected to MS identification and peptide mass fingerprinting (PFM). By searching the protein database, *α*-,*β*-, and *γ*-proteins, lens protein subunits, and associated active enzymes were identified. Among these, lens *β*A3, *β*B3, *γ*B, and *β*A4 structural proteins showed reduced expression in rats with D-galactose-induced cataracts, though normal levels were restored by intervention with syringic acid. Lens structural proteins of types *α*A, *α*B, *β*A2, *β*B1, *γ*N, and actin and ubiquitin carboxyl-terminal hydrolase L1 showed higher expression levels in the model group than in the normal group, but their expression levels decreased in the syringic acid groups. Endoplasm protein AR, associated with cellular metabolism in lens epithelial cells, was highly expressed in the model group, but its expression level was decreased in syringic acid groups. Based on these findings, it was proposed that AR may be a target protein for syringic acid.

### 3.4. Effect of Syringic Acid on Lens AR Gene Expression

**Figure 4:**
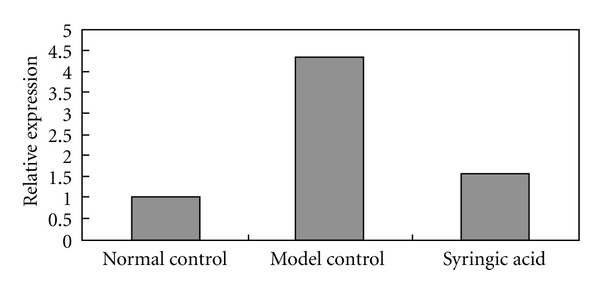
Effects of syringic acid (2%) on AR mRNA expression induced by 0.5% D-galactose. The expression of AR genes is normalized using the housekeeper gene (*β*-actin), which is evenly expressed in rat lens cells. Compared with the normal control group, mRNA levels of the AR gene in the model group increased 4.32-fold and 1.53-fold in syringic acid groups, respectively.

Compared with the normal control, expression levels of AR increased 4.32-fold in the D-galactose model group and 1.53-fold in the syringic acid group when analyzed by qRT-PCR (Figure 4). These findings likely indicate that syringic acid downregulates mRNA expression of AR.

### 3.5. Effect of Syringic Acid on AR Activity

Syringic acid inhibited AR activity in a concentration-dependent manner (Table 4). The IC_50_ was 213.17 *μ*g/mL. Data indicated that the inhibition of AR by syringic acid was noncompetitive and concentration dependent.

### 3.6. Molecular Binding of Syringic Acid with AR

**Figure 5:**
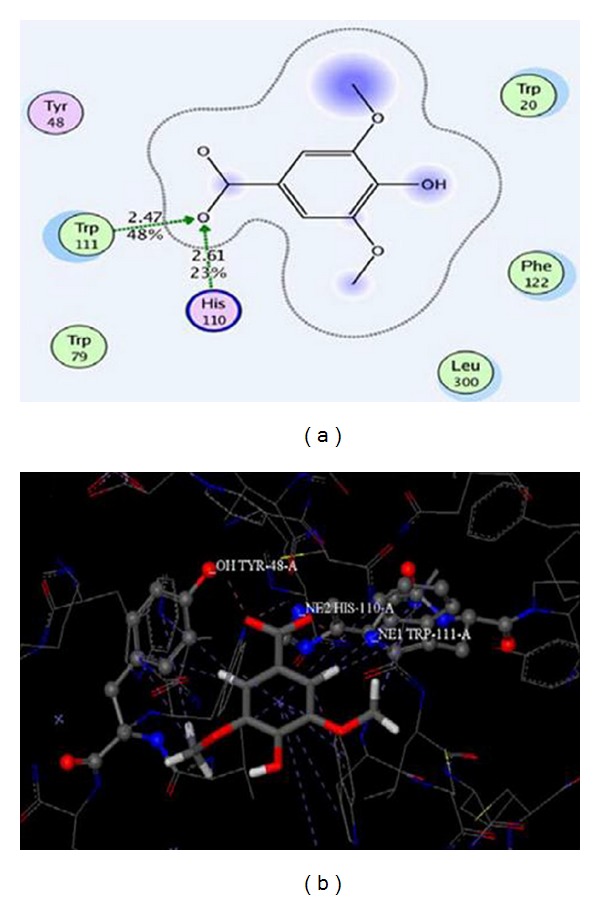
Binding of syringic acid to AR. (a) Residues surrounding the syringic acid and AR. (b) Three-dimensional (3-D) graphic syringic acid docking to AR. Syringic acid interacts with AR at the 7 amino acid residues Trp111, His110, Tyr48, Trp20, Trp79, Leu300, and Phe122. (a) Trp111 and His110 have the same hydroxyl ion (–OH) at position 1, constituting an intermolecular hydrogen bond. The 3-D graphic identified intermolecular hydrogen bonds between Tyr48 and O groups at position 2.

Ten docking conformations were recorded with minimal root mean square deviations (RMSDs), and the highest scores were subjected to modeling analysis. Previous studies have reported that hot spot residues of the AR inhibitor were Trp111, His110, and Tyr48 [24, 25, 27, 28]. Two-dimensional (2-D) graphics of the binding of syringic acid to AR identified interactions between the 7 amino acid residue Trp111, His110, Tyr48, Trp20, Trp79, Leu300, Phe122, and syringic acid (Figure 5). Of these, Trp111 and His110 have the same hydroxyl ion (–OH) at position 1, constituting an intermolecular hydrogen bond. The distance of these amino acids from the hydrogen bond was 2.47 Å and 2.61 Å, respectively.

No intermolecular hydrogen bonds were observed between Tyr48 and syringic acid using 2-D graphics. In the three-dimensional (3-D) graphics, however, intermolecular hydrogen bonds between Tyr48 and O groups were identified at position 2. These bonds are thought to play an important role in the binding of AR to inhibitors and the maintenance of the hydrophobic interaction between micromolecules and residues. Thus, it is proposed that the hydrogen bond and hydrophobic interaction between the aromatic ring and residue side chains act as the driving force for binding of syringic acid to AR.

**Figure 6:**
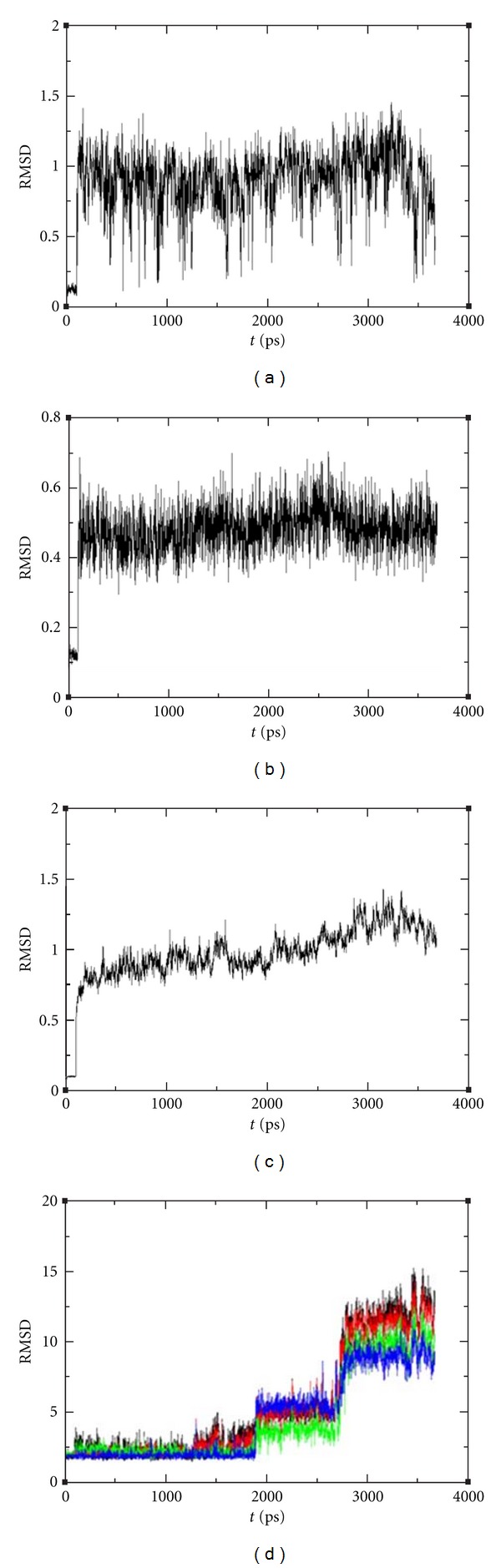
Dynamic simulations of syringic acid and AR. (a) Dynamics simulation trajectory of syringic acid and AR; (b) changes in NADPH within 4 ns; (c) changes in AR frame within 4 ns; (d) changes in hydrogen bonds between syringic acid and amino acid residues within 4 ns.

The results of dynamic simulation of syringic acid and AR after binding showed that the changes of RMSD within 4 ns were >1 and that the balanced phase at 4 ns was in the normal range (<3) (Figure 6(a)). The changes in coenzyme NADPH RMSD within 4 ns were >0.5, indicating a stable system (Figure 6(b)). The changes of the AR frame RMSD within 4 ns were <1.5, indicating a stable experimental protein frame (Figure 6(c)). The hydrogen bond formed after docking was stable within 2 ns, but changes in RMSD from 3-4 ns were >5. This observation suggests that the system is unstable (Figure 6(d)).

### 3.7. The Noncovalent Bonding Ratio between Syringic Acid and AR

Different molar ratios of syringic acid and AR formed syringic acid-AR complexes after binding. The relative molecular weight of syringic acid-AR complexes was analyzed by negative ion MS and deconvolution software (Table 5). The average molecular weights of syringic acid-AR complexes at a 27 : 1 molar ratio were then calculated using the obtained CSI-MS spectra.

**Figure 7:**
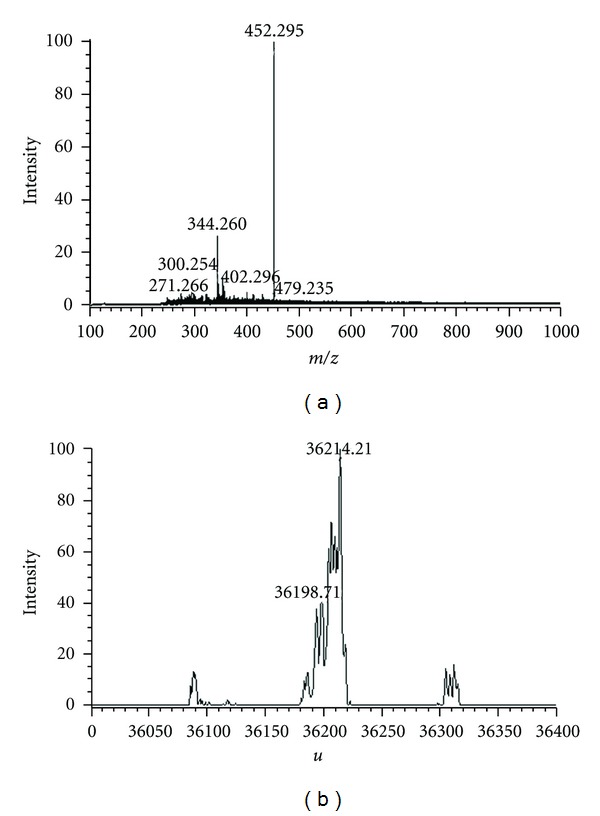
Mass spectra of relative molecular weights of the syringic acid-AR complex by molar ratios of syringic acid to AR. (a) Mass spectra for a molar ratio of syringic acid-AR of 27 : 1; (b) calculated average molecular weight of the syringic acid-AR complex.

When the molar weight of syringic acid is increased, the relative molecular weight of the syringic acid-AR complex gradually increased and then remained at its saturated relative molecular weight (Figure 7). This finding likely indicates that the binding of syringic acid to AR is concentration dependent.

The binding of syringic acid to AR was affected by pH and solution temperature. Different pH values were applied to the molar ratio of syringic acid to AR of 27 : 1 to investigate the formation of the syringic acid-AR compound at 37°C. The maximum molecular weight of 36206.79 was detected at pH 9, suggesting that changing pH values affects the binding of syringic acid and AR (Table 6). Different solution temperatures (5, 10, 15, and 25°C) were also used to investigate the formation of the syringic acid-AR compound at the same molar ratio (27 : 1) at pH 6.7. The maximum molecular weight of 36214.21 was detected at 10°C, suggesting that changing solution temperature affects syringic acid to AR binding (Table 7).

By detecting the molecular weight of the syringicacid-AR complex formed by binding different concentrations of syringic acid to 2.8 *μ*mol/L AR, the maximum relative molecular mass of the complex was calculated to be 36214.21. Further calculations also revealed that the maximum stoichiometric ratioofthe syringic acid-AR complex was 1 : 13.3.

## 4. Discussion

Syringic acid, one of phenolic constituents of *Herba Dendrobii*, was shown to inhibit diabetic cataracts in both *in vitro* lens cultures and *in vivo* rat models in the current study. Synergic acid forms a complex with AR that may reduce the occurrence and progression of diabetic cataracts caused by glycometabolism dysfunction. These cataracts are typically characterized by rapid development of lens cortex turbidity and posterior capsules in both eyes, and the condition cannot currently be treated without surgical intervention [29]. The number of diabetic patients worldwide is rising, accounting for 2% of the total global population and as much as 5% of the population in developed countries [30]. As diabetes, particularly Type 2 diabetes, becomes more prevalent, it is increasingly important to develop effective, nonsurgical treatments for diabetic cataracts.

The pathogenesis of diabetic cataracts is primarily associated with hyperglycemia-induced osmotic pressure disorder [31, 32]. In contemporary research, AR has been an important target enzyme in the search for antidiabetic cataract drugs. AR inhibitors (ARI) have been shown to be promising drugs for the prevention and treatment of diabetic cataracts in animal models [4, 5, 7], though significant liver and gastrointestinal side effects in humans still limit clinical applicability [6]. These early clinical experiences suggest if side effects can be effectively limited or managed, these compounds may improve future treatment for diabetic cataracts. 

Previous research has shown that apoptosis of human lens epithelial cells (HLECs) is the common cellular basis for various noncongenital cataracts [33]. D-galactose-produced experimental models for diabetic cataracts have been used to explore this condition in guinea pigs [34] and rats [35]. Using these well-established diabetic cataract models, the current study found that syringic acid intervention aided in maintenance of morphologic features of HLECs and protected lens cells *in vivo*. Interestingly, the optimal working concentration was found to be a medium dose of syringic acid (0.2 mg/mL). This study provides initial evidence for a potential antidiabetic cataract effect that merits further exploration.

To better understand the mechanism of syringic acid action, the total protein expression of lenses *in vivo* was studied using 2-DE. Lens *β*A3, *β*B3, *γ*B, and *β*A4 structural proteins evidenced reduced expression in rats with D-galactose cataracts. Intervention with syringic acid, however, was able to restore normal expression levels. Lens structural proteins *α*A, *α*B, *β*A2, *β*B1, *γ*N, actin, ubiquitin carboxyl-terminal hydrolase L1, and AR showed higher expression levels in control groups, but expression levels decreased when treated with syringic acid. Due to the well-documented association of AR with diabetic cataracts, AR is the most likely target protein for syringic acid. Other lens structural proteins and ubiquitin carboxyl-terminal hydrolase L1 may also be targets for syringic acid, though further studies are required to examine and confirm these potential mechanisms.

Both qRT-PCR findings and inhibition effects of syringic acid on AR activity support the hypothesis that AR is the most significant target protein for syringic acid. In qRT-PCR studies, syringic acid clearly downregulated mRNA expression for AR. Similarly, syringic acid was shown to inhibit AR in a noncompetitive and concentration-dependent manner. This dual mode of action of syringic acid at both the enzyme and mRNA levels offers potential benefits over previous agents that only targeted the enzyme level [5, 36]. Previous studies using experimental aldose reductase inhibitors have established the relationship between AR reductase inhibition and prevention of diabetic cataract progression. The specifically developed AR inhibitors KIOM-79 and GP-1447 were shown to be active in rat models of diabetic cataracts [4, 5]. These previous studies used either *in vitro*, *ex vitro*, or *in vivo* methods to investigate the effects of AR inhibition on diabetic cataract formation. Thus, the current study was designed to include a combination of both *in vitro* and *in vivo* experiments in order to provide a more complete understanding of the mechanism of AR inhibition. 

Syringic acid to AR binding was explored using molecular modeling to predict the interaction mode of these two molecules. Analysis of the resultant complex demonstrated that Trp111, His110, Tyr48, Trp20, Trp79, Leu300, and Phe122 are the primary amino acid residues involved in the binding process. This finding is in agreement with a previous study that showed the formation of stable hydrogen bonds by a natural phenolic marine inhibitor of aldose reductase with the hALR2 active pocket of residues Tyr48, His110, and Trp111 [24]. In addition to these 3 known binding residues, 4 other residues were identified on AR that are likely to be involved in syringic acid binding. Thus, the current research provides a more complete understanding of the AR and substrate complex binding process. 

Cold-spray ionization mass spectrometry (CSI-MS) and analysis of the negative ion source for the syringic acid-AR complex showed that the relative molecular weight of the syringic acid-AR complex was related to the electrical current carrying capacity of syringic acid. The molecular weight of the complex was highest when the molar ratio of syringic acid to AR was 27 : 1. Increasing the molar weight of syringic acid to even higher levels resulted in a gradual decrease in binding. These results also demonstrate that binding of syringic acid to AR is both temperature and pH dependent, reaching optimal binding levels at 10°C and pH 9.0. By documenting these details of syringic acid-AR complex binding conditions, this study provides the groundwork for further exploration and development of syringic acid as a potential ARI for treatment of diabetic cataracts. 

Future studies will be needed to investigate the potential toxicity of syringic acid derived from *Dendrobium* in other organs and systems. Additionally, further studies will be required in order to investigate its specificity for diabetic cataract tissues and to explore related side effects of this treatment. Due to the well-documented historical use of *Dendrobium* extracts in traditional Chinese medicine for vision treatment, there is strong potential for use without significant side effects; however, further studies will be required to assess potential side effects that occur as dosage and concentrations increase beyond those found in natural extracts. Further investigation of the therapeutic role of syringic acid on common cataracts, not those caused by diabetes, may also merit further study based on these preliminary findings.

Cumulatively, the current study provides the first definitive confirmation of syringic acid as the effective working component in *Herba Dendrobii*, demonstrating that it is capable of protecting rats from developing experimentally induced diabetes cataracts both *in vitro* and *in vivo*. Further investigation of the mechanism of syringic acid action revealed that it downregulated mRNA expression of AR and inhibited AR activity in a noncompetitive and dosage-dependent manner. Thus, syringic acid is able to exert a physiological effect on glucose metabolism and cataract formation. Characteristics of the AR syringic acid complex were identified in terms of binding residue, binding ratio, pH, and optimal solution temperature. Documentation of these parameters provides a useful basis for further development of syringic acid for potential future use in therapeutic management of diabetes cataracts in humans.

## Supplementary Material

Qualitative and Quantitative Analysis of Syringic Acid Extracted from Dendrobium.

## Figures and Tables

**Table 1:** Effect of syringic acid at different concentrations on the proliferation of HLEC.

Group	Syringic acid concentration (g/L)	HLEC proliferation	Survival rate (%)	Inhibition ratio (%)
Normal control	—	0.5545 ± 0.0029	100	0
Model control	—	0.2606 ± 0.0029*	47.01*	52.99*
High-dose syringic acid	0.4	0.4720 ± 0.0032	74.38	25.62
Medium-dose syringic acid	0.2	0.5250 ± 0.0013**	79.97**	20.30**
Low-dose syringic acid	0.1	0.4610 ± 0.0035	71.67	28.33

*P < 0.05 compared to syringic acid groups and normal control group.

**P < 0.05 compared to high-dose and low-dose syringic acid groups.

Data were expressed as mean ± SD (*n* = 6) or percentages. Inter-group differences were analyzed using the Nemenyi multiple-comparison *H* test.

**Table 2:** Opacification scores, expressed as total number of plus (+) signs, of cultured lenses in different groups at various time points *in  vitro*.

Groups	N*	6 h	12 h	18 h	24 h
Normal control	5	0	0	0	0
Model control	5	5	9	13	15
Catalin control	5	0	4**	7**	9**
Syringic acid groups					
High dose	5	3	6	7	11
Medium dose	5	1	2^#^	3^#^	6^#^
Low dose	5	3	7	11	12

N*: number of animals used in each group.

**P < 0.05 compared to all other groups.

^#^ *P* < 0.05 compared to the high- and low-dose syringic acid groups.

Intergroup differences were analyzed using the Nemenyi multiple-comparison *H* test.

Clear lines indicated Grade 0 turbidity; vague lines with a distinguished outline indicated Grade I (+) turbidity, a vague outline with indistinct line centers indicated Grade II (++) turbidity, and a cloudy cortex with all lines invisible indicated Grade III (+++) turbidity. (Numbers in columns from 6 h to 24 h are the summed “+” for each group, representing the general turbidity.)

**Table 3:** Comparison of lens turbidity by group at different time points after medication.

Groups	N*	20 days	30 days	40 days	60 days	90 days
Normal control	40	0	0	0	0	0
Model control	40	77	92	98**	110**	130**
Catalin control (0.8 mg/15 mL)	40	77	92	65	33	23
Syringic acid (2%)	40	77	92	57^#^	29^#^	15^#^

N*: number of eyes used in each group.

**P < 0.01 compared with the normal control and other groups.

^#^ *P* < 0.05 compared with Catalin control.

Intergroup differences were analyzed using the Nemenyi multiple comparison *H* test.

Clear lines indicated Grade 0 turbidity; vague lines with a distinguished outline indicated Grade I (+) turbidity, a vague outline with indistinct line centers indicated Grade II (++) turbidity, and a cloudy cortex with all lines invisible indicated Grade III (+++) turbidity. (Numbers in columns from 20 d to 90 d are the summed “+” for each group, representing the general turbidity.)

**Table 4:** Inhibition of AR activity by syringic acid.

Syringic acid (*μ*g/mL)	Inhibition ratio (%)
5	11.728
10	30.740
100	52.970
200	68.630

Syringic acid inhibited AR activity in a noncompetitive and concentration-dependent manner (IC_50_ = 213.17 *μ*g/mL).

**Table 5:** Molecular mass of syringic acid-AR complex by molar ratio of syringic acid and AR.

Syringic acid	AR molecular mass
3 : 1	36196.69
9 : 1	36206.79
27 : 1	36214.21
81 : 1	36202.75
243 : 1	36206.79
729 : 1	36198.04

**Table 6:** pH value effects on syringic acid and AR binding.

pH value	Mr of syringic acid-Ar compound
6.0	36197.38
6.7	36202.75
8.0	36204.77
9.0	36206.79

The maximum molecular weight of 36206.79 was detected at pH 9, suggesting that changing pH values affects syringic acid and AR binding.

**Table 7:** Solution temperature effects on syringic acid and AR binding.

Tm (°C) compound	Mr of syringic acid-Ar compound
5	36203.43
10	36214.21
15	36197.36
20	36199.38

The maximum molecular weight of 36214.21 was detected at 10°C, suggesting that changes in solution temperature affect syringic acid and AR binding.
