# Supplementary material for: Syringic Acid Extracted from Herba dendrobii Prevents Diabetic Cataract Pathogenesis by Inhibiting Aldose Reductase Activity
Source: Evid Based Complement Alternat Med. 2012 Dec 29;2012:426537. doi: 10.1155/2012/426537 (PMC3545393; doi:10.1155/2012/426537)
Supplement: Supplementary file 1 — Qualitative and Quantitative Analysis of Syringic Acid Extracted from Dendrobium. [file 426537.f1.doc]

**Qualitative and Quantitative Analysis of Syringic Acid Extracted from Dendrobium**

**1. Identification of syringic acid extracted from Dendrobium by TLC**

- 1. **TLC chromatography conditions.** Thin layer board: silica gel G thin layer board; Developing agent: Petroleum ether-ethyl acetate（3:1）
  2. **Reference substance solution preparation** Take standard syringic acid (S9484, Ruibio, Germany) and methanol made every 1 mL contains 0.1 mg of solution, as reference substance solution.
  3. **The product solution preparation** Precision takes sample amount, with methanol solution, match into 0.2 mg/mL solution.
  4. **Expansion and color** Take petroleum ether, ethyl acetate (3:1) about 10mL to double slot open box, preliminary equilibrium 15 min, expansion, remove and dry, exhibition from 8 cm. Uniform spraying 2% sulfuric acid vanillin solution, color to the spots were clear. Rf value was 0.33.

**1.5 Results** The fuchsia spots of dendrobium-extracted syringic acid were the same as the standard syringic acid.(fig.1).


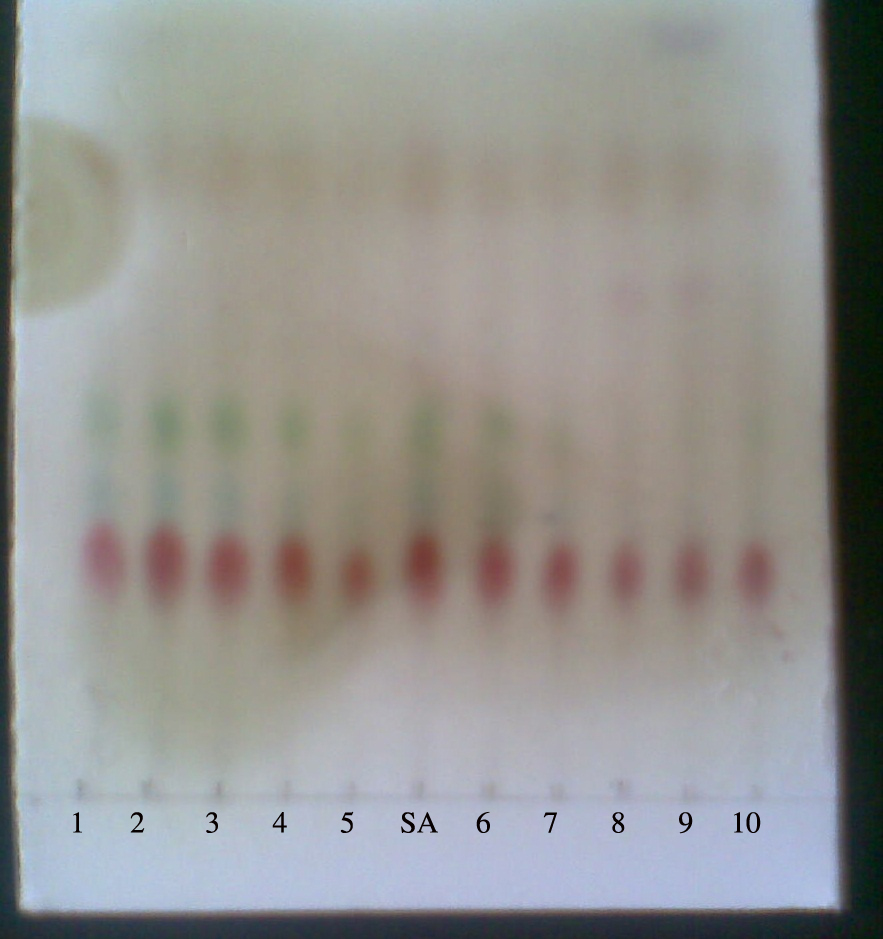


Figure 1 TCL Chromatograms of standard syringic acid (SA) and dendrobium-extracted syringic acid (1-10).

1. **Syringic acid purity determination by HPLC**
   1. **Chromatographic condition** The RP–HPLC analyses were performed with Shimadzu LC – 20A liquid chromatograph and equipped with a TSKgel ODS-100V (RP C18 column size 4.6mm × 250mm; particle size, 5 μm). Mobile phase were acetonitrile: 0.2% formic acid (35:65) and detection wavelength was 280 nm . The column temperature was 30℃,and the velocity was 1 mL/min.

**2.2 Syringic acid standard curve preparation.**

Precision takes standard syringic acid right amount to 1 mg/mL made methanol solution, as reference substance solution. Precision takes the solution (1, 2, 3, 4, 5mL) to 10 ml volumetric flask, with methanol constant volume to scale. Above the concentration of syringic acid solution were injected 10μL to liquid chromatograph. With concentration for x axis, the corresponding peak area for ordinate, calculated the regression equation. y=4×10^6^x + 89644(r=0.9969), linear range: 0.05mg-0.6mg (fig.2).

Figure 2 The standard curve of standard syringic acid

**2.3 The retention time of the major peaks of samples(dendrobium-extracted syringic acid) and standard syringic acid are consistent.(fig.3)**


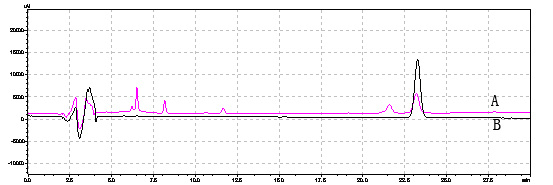


(A samples B standard syringic acid)

Figure 3 HPLC chromatograms of standard syringic acid (B) and samples (A)

**2.4 Take the same precision experiment**

Selected product solution, continuous sample 5 times, measured RSD of 0.49%.

**2.5 Repetitive experimental**

Take the same batch samples of 6; the method as sample content determination. The RSD of syringic acid was 1.19%.

**2.6 Stability testing**

Take the same samples respectively in 0, 1, 3, 6, 12, 16, 24, 48,72 h measurement, the results have no obvious difference (RSD=0.42%, n=9) and syringic acid was stabile in 72 h.

**2.7 Sample purity determination**

Precision takes sample amount, with methanol solution, match into 0.2 mg/ml solution. Precision absorb change solution 10 ul, injection liquid chromatograph. The peak area was 846167. Results showed that samples contain syringic acid 98%.
